# Associations between screen use, outdoor time/daylight exposure and sleep changes during the first COVID‐19 lockdown in French children from the ELFE and EPIPAGE2 birth cohorts

**DOI:** 10.1111/cns.14128

**Published:** 2023-02-20

**Authors:** Alex Wilfried Kamga Fogno, Alexandra Rouquette, Claude Gronfier, Jonathan Y. Bernard, Sabine Plancoulaine, SAPRIS study group

**Affiliations:** ^1^ Université Paris Cité and Université Sorbonne Paris Nord, Inserm, INRAE, Center for Research in Epidemiology and StatisticS (CRESS) Paris France; ^2^ Université Paris‐Saclay, UVSQ, Inserm U1018, CESP Paris France; ^3^ AP‐HP Paris‐Saclay, Hôpital du Kremlin Bicêtre, Service de Santé Publique et d'Epidemiologie Le Kremlin Bicêtre France; ^4^ Université Claude Bernard Lyon 1, INSERM, CRNS, Centre de Recherche en Neurosciences de Lyon CRNL U1028 UMR5292 Bron France; ^5^ Singapore Institute for Clinical Sciences (SICS), Agency for Science, Technology and Research (A*STAR) Singapore Singapore

**Keywords:** children, COVID lockdown, outdoor light exposure, screen, sleep

## Abstract

**Aims:**

To investigate associations between outdoor and screen time and changes in sleep patterns in children from two nationwide birth‐cohorts in the SAPRIS project.

**Methods:**

During the first French COVID‐19 pandemic lockdown, volunteer parents of children enrolled in the ELFE and EPIPAGE2 birth‐cohorts completed online questions about their child's outdoor time, screen time, and changes in sleep duration and quality compared with the pre‐lockdown situation. In 5700 children (aged 8–9 years, 52% boys) with available data, we assessed associations between outdoor time, screen time, and sleep changes using multinomial logistic regression models adjusted for confounders.

**Results:**

Children spent on average 3 h08 outdoors and 4 h34 using screens/day (3 h27 for leisure, 1 h07 for class‐work). Sleep duration increased in 36% of children and decreased in 13.4%; sleep difficulties appeared/increased in 22.5% and decreased/disappeared/remained stable in 18.3%. After adjustment, increased screen time, especially for leisure, was associated with increased and decreased sleep duration (OR(95%CI) = 1.03(1.00–1.06) and OR = 1.06(1.02–1.10), respectively). No association was observed between outdoor time and sleep changes after adjustment.

**Conclusions:**

Our study adds evidence for the association between high leisure‐time screen time and shorter sleep time. It supports current screen guidelines for children, especially during leisure time and for those whose sleep duration is short.

## Graphical abstract

Among 5700 children aged 8–9 years from two French birth‐cohorts, 1/5 experienced new/increased sleep difficulties and about 1/7 of them decreased their sleep duration during the first COVID‐19 pandemic lockdown. Higher screen time, especially for leisure, was associated with shorter sleep duration. No association was observed between outdoor time and sleep changes. This supports current screen time guidelines for children, especially for leisure and for those who have short sleep durations.

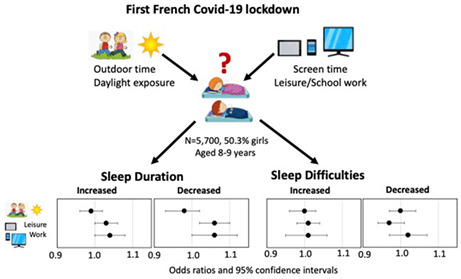

## 1 INTRODUCTION

Sleep is essential for human life. Circadian sleep–wake rhythmicity is associated with the regulation of many functions, including metabolic, hormonal, cardiorespiratory, immune, neurological, cognitive, and psychological functions.^1^, ^2^, ^3^, ^4^ It is largely regulated by light, via the stimulation of melanopsin ganglion cells and the suprachiasmatic nucleus of the hypothalamus, resulting in the inhibition of melatonin secretion and activation of the wakefulness systems.^5^ These effects depend on (i) the light spectrum (wavelengths), with a maximum effect around 480 nm (blue light from LED bulbs, computer screens or smartphones); (ii) the light intensity; (iii) the exposure duration, and (iv) the exposure period, with a maximum effect at bedtime and at dawn.^6^, ^7^ Thus, inappropriate light exposure (less exposure to outdoor light or overexposure to blue light, especially in the evening) could lead to disturbances of the circadian rhythm and therefore of sleep.^8^, ^9^, ^10^ Sleep disturbances in children are associated with current and subsequent health, behavioral, and cognitive difficulties.^11^, ^12^, ^13^, ^14^

On December 31, 2019, the first cases of COVID‐19 were reported in Wuhan, China. A few months later, this disease became a pandemic, with 87,137 cases listed worldwide as of March 1, 2020.^15^ This unprecedented situation led to lockdown measures ‐ sometimes successive ‐ in many countries around the world. In France, the first strict general lockdown, including school closures, was declared on March 16, 2020 and ended on May 11, 2020. This was followed by a period of restrictions, still including school closures, travel restrictions and a ban on gatherings of more than 10 people until June 2. These periods changed daily behaviors and activities, which had effects on exposure to outdoor light and screens and on sleep patterns.

Several studies reported an increase in both qualitative and quantitative sleep difficulties during lockdown in adults.^16^, ^17^ In preschoolers, evidence is less consistent. Some studies report shorter sleep duration and lower quality, and others the inverse.^18^, ^19^, ^20^ In school‐age children and adolescents, studies show a reduced sleep quality, with an alteration in circadian synchronization, and an increased sleep duration.^21^, ^22^ In France, sleep durations are longer than in other countries with similar socioeconomic status,^23^, ^24^ so the impact of lockdown on sleep durations and difficulties may be different. In addition, very few studies have examined the associations between screen use, outdoor time/daylight exposure, and sleep in children.

In this study, we aimed first at describing school‐aged children's behaviors, their changes during the lockdown, particularly regarding (i) changes in sleep duration and sleep difficulties, (ii) exposure to screen use (overall time and by purpose of use), and (iii) outdoor time/daylight exposure; and second at analyzing the associations between screen use, outdoor time/daylight exposure, and sleep changes during the first French lockdown.

## 2 SUBJECTS AND METHODS

### 2.1 Study participants

Parents and children included were those recruited in 2011 from the ELFE^25^ and EPIPAGE‐2^26^ birth cohorts and who agreed to participate in the SAPRIS project (“SAnté, Perception, pratiques, Relations et Inégalités Sociales en population générale pendant la crise COVID‐19”), during the first lockdown in spring 2020.^27^ Briefly, ELFE is a nationwide, multidisciplinary, birth cohort study, which included 18,329 children born in a random sample of 349 maternity units in mainland France in 2011. Inclusion criteria were as follows: singleton or twins born after 33 weeks of gestation, to mothers aged 18 years or older. EPIPAGE‐2 is a population‐based prospective study which included all infants live born or stillborn and all terminations of pregnancy between 22 and 31 completed weeks of gestation in all the maternity units in 25 French regions, with an additional sample of moderate preterm births, i.e., births and late terminations at 32–34 weeks, included in the same regions. In both cohorts, children were regularly followed‐up. In total, 6193 children participated in the SAPRIS project among the 16,059 ELFE and EPIPAGE‐2 families solicited. We excluded every other twin to avoid family clusters (*N* = 228) and children with missing sleep data (*N* = 265). A total of 5700 children aged 8–9 years old were included in the analysis, 4683 and 1017 from the ELFE and EPIPAGE‐2 cohorts, respectively.

In both cohorts, mothers provided written consent for their own and their child's participation at inclusion. Fathers signed the consent for the child participation when present on inclusion days or were informed about their rights to oppose. Regulatory authorities overseeing ethical data collection in France approved ELFE and EPIPAGE‐2 (Comité de Protection des Personnes (respectively, CPP n°IDFIX‐11‐024, CPP SC‐2873); Comité National Informatique et Libertés (CNIL *n*°910,504, CNIL *n*°91,009), and CNIS *n*° 2011X716AU for ELFE, CCTIRS *n*°10.626 for EPIPAGE‐2).

### 2.2 Data

Data were collected in the SAPRIS project from parents through two internet questionnaires. The first questionnaire was available from April 16 to May 4, 2020 (T1) and the second one from May 5 to May 31, 2020 (T2). Information on sleep and light exposure was asked only once at either T1 or T2. We considered the responses collected at T1 and those at T2 when missing at T1.

#### 2.2.1 Sleep and light exposure

Collected sleep data focused on change in both sleep duration and sleep difficulties during the lockdown compared to before. The questions asked to the parents were: “Would you say his or her sleep duration has changed since the lockdown began?” (No, it is as usual; yes, it has increased; yes, it has decreased), and “Would you say that since the lockdown began, your child has had difficulty sleeping (such as difficulty falling asleep, waking up at night frequently or too soon with no opportunity to fall back asleep)?” (That have appeared; that have increased; that have decreased; that have disappeared, that have remained stable; no difficulty). We grouped the categories “that have appeared” and “that have increased” in one category “Appeared/increased” and the categories “that have decreased,” “that have disappeared,” and “that have remained stable” (1.4% of the children) into one category “Stable/improved”.

Outdoor time/daylight exposure was estimated from two questions about the last 7 days, “How much time per day did your child spend playing sports or walking outside home?” and “How much time per day did your child spend on physical activities in the home yard or garden?” Total outdoor time/daylight exposure was calculated in hours and minutes. Screen exposure was estimated from four questions, “How much time per day did your child spend on console or online games?”, “How much time per day did your child spend on social networks (Facebook, Instagram, Snapchat, etc.)?”, “How much time per day did your child spend on television or other screens for school or educational programs?”, and “How much time per day did your child spend on television or other screens for other programs?” Total screen time, screen time for schoolwork and for leisure were calculated in hours and minutes. Because outliers with total screen time > 24 h/day were observed, we censored the values >8 h/day for each item, and censored the children with a total screen time > 24 h/day (*n* = 470, 8.2%).

#### 2.2.2 Confounding factors

Confounding factors were selected based on both the literature and a directed acyclic graph (DAG).^28^ The considered socioeconomic factors were: maternal education (bachelor's degree, bachelor's degree +2 years, > bachelor's degree +2 years), household socio‐professional category (upper and middle management; employed or self‐employed; blue‐collar and inactive), family structure (2 parents, 1 parent or alternating custody), single child (no, yes), and maternal age; more specifically during this lockdown period: household financial status (affluent, income constant; affluent, income decreasing; modest, income constant; modest, income decreasing), work status within the couple (neither works; one teleworks, one does not; at least one works outside the home; both are teleworking), dwelling with garden or yard (no, yes), living area (rural, urban), dwelling occupancy index calculated as the ratio of the number of rooms to the number of persons living in the dwelling. Children’ factors were gender, birth term, presence of a chronic or developmental pathology (no, yes), time of physical activity indoors during the survey (hour/day), overall behavior assessed by the Strengths and Difficulties Questionnaire (SDQ), and the presence of at least one of the following symptoms in the last 15 days: fever, unusual fatigue, muscle aches/pains, breathing difficulties/unusual shortness of breath, runny nose, pharyngitis, sore throat, febrile conjunctivitis, trouble smelling or tasting, nausea/vomiting, diarrhea, chest pain/oppression, appearance of persistent patchy frostbite on feet, hands, or face. The cohort (ELFE, EPIPAGE 2) and the wave of the questionnaire (T1, T2) were also taken into account.

### 2.3 Statistical analysis

Analyses were performed using SAS® version 9.4 software (SAS Institute Inc., Cary, North Carolina). Comparison of the characteristics of children included and excluded from the analysis was performed with Chi^2^ tests for categorical variables, and Student *t*‐tests for continuous quantitative variables. Wilcoxon‐Mann–Whitney rank test was used when frequency distribution was nonnormal.

Multiple imputations of missing data were performed by using Fully Conditional Specification (FCS). Binary variables were imputed by logistic regression, nominal or ordinal variables were imputed by multinomial logistic regression, and continuous variables by linear regression. 20 databases were imputed and analyzed. Estimates and confidence intervals were pooled to obtain overall results. Multinomial logistic regression models, with either stable sleep time or no sleep difficulty as reference categories, were used to study the associations between the duration of exposure to light, whether outdoor or via screens, and the different sleep changes, without (M1) and with (M2) adjustment for family, child, and study characteristics. Interactions between outdoor light and screen exposure times were tested by adding multiplicative terms into the models.

## 3 RESULTS

Compared to the children aged 8–9 years old followed in the SAPRIS project and excluded from our analysis (*n* = 493), the included children (*n* = 5700) were more often from the ELFE cohort, from households with both parents having high socioeconomic level, working during the lockdown at home or outside and living in large dwelling (occupancy index >1). (Table 1).

**TABLE 1:** Population description.

	Non‐included	Included	Missing data
	(*N* = 493)	(*N* = 5700)	Included sample
	% (*N*) or Median (Q1‐Q3)	% (*N*) or Median (Q1‐Q3)	N
Study			
Cohort			0
ELFE	44.8 (221)	82.2 (4683)	
EPIPAGE 2	55.2 (272)	17.8 (1017)	
Questionnaire timing			0
T1	74.7 (368)	83.9 (4782)	
T2	25.4 (125)	16.1 (918)	
Child characteristics			
Sex (girl)	50.3 (244)	48.9 (2768)	44
Single child	18.5 (59)	18.6 (1055)	26
Gestational age (weeks)	34 (30–39)	39 (38–40)	76
Familial characteristics			
Maternal age (years)	30 (27–34)	31 (28–34)	61
Education			60
<bachelor	30.9 (149)	22.6 (1273)	
bachelor +2 y	26.7 (129)	25.0 (1411)	
>bachelor +2 y	42.4 (205)	52.4 (2956)	
Single parenting	20.9 (93)	13.9 (794)	0
Household socio‐professional category			115
upper and middle management	49.3 (190)	56.7 (3165)	
employed or self‐employed	36.9 (142)	33.8 (1890)	
blue‐collar and inactive	13.8 (53)	9.5 (530)	
Work status within the couple			12
neither works	64.0 (220)	34.2 (1944)	
one teleworks, one does not	10.8 (37)	37.1 (2110)	
at least one works outside the home	20.6 (71)	22.1 (1257)	
both are teleworking	4.6 (16)	6.6 (377)	
Household financial status			28
affluent, income constant	55.7 (157)	57.0 (3232)	
affluent, income decreasing	9.2 (26)	9.9 (563)	
modest, income constant	18.4 (52)	16.1 (912)	
modest, income decreasing	16.7 (47)	17.0 (965)	
Dwelling with garden or yard	92.1 (339)	91.7 (5212)	14
Dwelling occupancy index			30
>1	55.6 (178)	61.67 (3490)	
1	26.6 (85)	27.0 (1532)	
<1	17.8 (57)	11.4 (648)	
Living area (rural)	40.6 (129)	43.0 (2439)	34

The median and mean outdoor time/daylight exposure time were 2 h03 (Q1‐Q3: 1 h26‐3 h53) and 3 h08 (95%CI: 2 h58‐3 h17) per day, respectively, at age 8–9 years old. Median and mean screen time (any kind and any use) were 3 h43 (Q1‐Q3: 2 h25‐5 h55) and 4 h34 (95%CI: 4 h28‐4 h39) per day. Median and mean screen time for leisure represented 2 h56 (Q1‐Q3: 1 h44‐4 h14) and 3 h27 (95%CI: 3 h15‐3 h31), while median and mean screen time for schoolwork represented 0 h29 (Q1‐Q3: 0 h00‐1 h09), 1 h07 (95%CI: 1 h04‐1 h09). Sleep duration was modified for 49.4% of children: it increased and decreased for 36.0% and 13.4% of them, respectively. During this first period of lockdown, 59.2% of the children had no sleep difficulties, 18.3% had resolved, decreased or unchanged sleep difficulties. Sleep difficulties appeared or increased for 22.5% of the children (i.e., 10.4% and 12.1% of the children, respectively).

Unadjusted analyses showed a negative association between outdoor time/daylight exposure time and decreased sleep duration and conversely a positive association between all screen times and both increased and decreased sleep durations (Table 2). The associations between total screen time and sleep durations persisted after accounting for confounders, while, when considering screen time according to the purpose of the use, the only associations remaining were those between screen time for leisure and sleep duration changes, the effect being stronger with decreased sleep duration (OR = 1.06 95% CI (1.02–1.10), *p* = 0.002).

**TABLE 2:** Raw and adjusted associations between both outdoor time/daylight exposure and screen use (total, for leisure and school‐work), and sleep changes during the first lockdown (*N* = 5700). All durations are in hours/day.

	Sleep duration			Sleep difficulties		
	Stable	Increased	Decreased	None	Appeared/Increased	Stable/Improved
	*N* = 2884	*N* = 2052	*N* = 764	*N* = 3374	*N* = 1283	*N* = 1043
	OR (95%CI)	OR (95%CI)	OR (95%CI)	OR (95%CI)	OR (95%CI)	OR (95%CI)
Total screen time						
M1. Unadjusted associations						
Outdoor time/daylight exposure	Ref	0.99 (0.96–1.02)	0.96 (0.93–1.00)	Ref	0.95 (0.93–0.98)	0.99 (0.96–1.02)
Screen time	Ref	1.04 (1.02–1.06)	1.09 (1.06–1.12)	Ref	1.04 (1.02–1.06)	1.00 (0.98–1.03)
M2. Adjusted associations†						
Outdoor time/daylight exposure	Ref	0.99 (0.96–1.02)	0.98 (0.93–1.02)	Ref	1.00 (0.96–1.04)	1.00 (0.97–1.04)
Screen time (h/d)	Ref	1.03 (1.01–1.05)	1.06 (1.03–1.09)	Ref	1.02 (0.99–1.05)	0.99 (0.96–1.01)
Purpose‐specific screen time						
M1. Unadjusted associations						
Outdoor time/daylight exposure	Ref	0.99 (0.96–1.02)	0.96 (0.92–1.00)	Ref	0.96 (0.93–0.99)	0.99 (0.96–1.02)
Leisure screen time	Ref	1.04 (1.01–1.06)	1.09 (1.05–1.12)	Ref	1.03 (1.00–1.06)	0.98 (0.95–1.01)
School screen time	Ref	1.05 (1.01–1.09)	1.09 (1.03–1.15)	Ref	1.04 (0.99–1.09)	1.03 (0.98–1.08)
M2. Adjusted associations†						
Outdoor time/daylight exposure	Ref	0.99 (0.96–1.02)	0.98 (0.93–1.02)	Ref	1.00 (0.96–1.03)	1.00 (0.97–1.04)
Leisure screen time	Ref	1.03 (1.00–1.06)	1.06 (1.02–1.10)	Ref	1.01 (0.98–1.04)	0.97 (0.94–1.01)
School screen time	Ref	1.04 (1.00–1.08)	1.06 (1.00–1.12)	Ref	1.01 (0.95–1.06)	1.02 (0.97–1.07)

*Note*: † Adjusted for family characteristics (maternal education, household socio‐professional category, family structure, single child, maternal age, household financial status, work status within the couple, dwelling with garden or yard, living area, dwelling occupancy index), children characteristics (gender, birth term, presence of a chronic or developmental pathology, indoors physical activity duration, SDQ, presence of infectious symptoms), the cohort and the wave of the questionnaire.

Unadjusted analyses also showed negative associations between outdoor time/daylight exposure time and appeared/increased in sleep difficulties, and a positive association between both total screen time and leisure screen time and appearance/increase of sleep difficulties. However, these relations no longer remained after adjusting for confounders.

## 4 DISCUSSION

About one third (35%) of the children included in our study sample increased their sleep duration and more than half of them showed a stable sleep duration during the first lockdown in France in March–May 2020. This is in line with an international cross‐sectional study, including 392 children aged 6–13 years from different continents during lockdown, that reported specifically an increased and stable sleep durations in 30% and 50% of the European children, respectively.^29^ Globally, studies that have compared sleep duration collected by parental questionnaires before and during lockdown showed an overall increase in sleep duration in school‐aged children.^30^, ^31^, ^32^, ^33^, ^34^, ^35^ When quantified, this increase varied from 17 to 39 min per night^30^, ^33^ or a doubling of the proportion of children sleeping more than 10 h per night,^32^ the recommended sleep duration for school aged‐children this age range.^36^ These studies reported bedtime and wake‐up time shift, probably facilitated by reduced school and/or transportation constraints.^29^, ^30^, ^31^, ^32^, ^33^, ^34^ We reported sleep difficulties' appearance or increase in 22% of the children. This deterioration in sleep quality during lockdown has been reported in school‐aged children,^32^, ^37^ for example, in Italian children aged 6 to 13 years, longer sleep latency, more frequent nocturnal awakenings, and nightmares in 27%, 8% and 16% of children, respectively.^32^ Unfortunately, the question asked on sleep difficulties in the SAPRIS survey did not allow distinction between the different reasons of the difficulties.

Lockdown limits, by definition, movements outside homes. In France, during the first lockdown in March–May 2020, going outside homes was allowed for only 1 h per day to run essential errands or take out pets. However, we reported a mean outdoor time/daylight exposure of children of 3 h per day, i.e., three times the authorized duration and much more than the average outdoor time or outdoor play duration for the age range reported in a recent systematic literature review (i.e., in habitual life settings and out of the context of lockdown).^38^ Indeed, the reported average duration of outdoor time and outdoor play for children aged 3 to 10 years were between 42 and 240 min per week (i.e., 6 min to 34 min/day) and between 2 h00 and 10 h18 per week (i.e., 17 min to 1 h28 per day), respectively.^38^ A Canadian study, conducted during lockdown, showed that children living in a house with access to nearby play areas were associated with increased outdoor activities.^39^ Thus, high outdoor time/daylight exposure during the lockdown might be explained by the high availability of a private or collective yard or garden in the current study (92%). Indeed, the associations observed between increasing outdoor time/daylight exposure and lower odds for both decreasing sleep duration and sleep difficulties were accounted for by confounders including having home access to outdoor areas.

The mean and median screen time in the studied French children (4 h34 h and 3 h43 per day, respectively) are lower than those reported in the USA, Italian, or worldwide school‐aged children. Indeed, North‐American children used screen for a mean duration of 3 h40 during lockdown,^31^ Italian school‐aged children (6–17 years, mean 13 years) for 7 h39,^33^ and 69% of those Italian children aged 12–13 years were exposed to screens for more than 3 h per day.^32^ This prevalence raised 83% of children from different continents (Americas, Middle East, and Europe) aged 6 to 12–13 years were exposed to screens for more than 3 h per day.^29^ The difference might be explained by the large opportunity for the studied children to access outdoor activities within a private or collective yard or garden. This information is not reported in the other studies. One important limitation of our study is that the screen use questionnaire was not validated and suffered from measurement bias, with a substantial number of invalid screen time (i.e., daily screen time greater than 24 h per day) that we excluded from our analyses.

Each hour of screen use, regardless the reason (leisure or school/work), was associated with an increased odds of sleep duration change and sleep difficulties. However, the associations between screen time for school work and sleep duration modifications were accounted for by confounding factors as well as the associations between screen time, regardless of the reason, and sleep difficulties. Several studies reported increases ranging from 45 min to 4.5 h per day of screen time during the lockdown in primary school children,^29^, ^30^, ^31^, ^33^ due, in particular, to a greater proportion of time spent on leisure activities, including social networks.^29^ Those studies also reported association between increased screen time and decreased sleep time during lockdown.^29^, ^30^ We report here that each hour of leisure screen time was associated with increased odds of decreased sleep duration compared to before the lockdown. Screen for leisure is also highly consumed in the evening,^40^, ^41^ since up to 75% of children aged 6–17 years have screens in their bedroom.^42^ It is now recognized that exposure to blue light from screens in the evening blocks the secretion of melatonin, a hormone that promotes sleep.^6^, ^43^ In addition, excessive screen time, especially before bedtime, has been shown to promote a phase delay of the circadian timing system (later bedtime and later rising) and difficulties in falling asleep.^8^, ^43^, ^44^, ^45^ In our study, we did not have information on the timing of screen use and therefore cannot confirm that these non‐visual effects of light were involved in the decreased sleep duration observed, however, they cannot be excluded. Especially because we showed that screen use for school‐work, usually done during daytime, was not associated with any sleep modification either quantity or quality after accounting for confounders. Other non‐exclusive and possibly cumulative mechanisms may exist and could explain the observed associations, such as the displacement of sleep time by screen time and the stimulation of cognitive activity caused by the content of the media viewed.^46^

In conclusion, this study shows that screen time, especially for leisure, was associated with sleep duration modifications during lockdown. It supports current screen guidelines for children, especially for leisure and for those whose have sleep troubles.

## AUTHOR CONTRIBUTIONS

Alex Wilfried Kamga Fogno performed the analyses and wrote the first draft of the manuscript. Alexandra Rouquette designed the data collection, Claude Gronfier designed the study, Jonathan Y. Bernard and Sabine Plancoulaine designed the study and supervised the analyses. All co‐authors were involved in both interpretation and critical revision of the manuscript. All co‐authors approved the final version.

## CONFLICT OF INTEREST STATEMENT

No interest to disclose. The authors have no conflict of interest.

## Data Availability

The data underlying this article cannot be shared publicly due to the privacy of individuals that participated in the study. The data will be shared on reasonable request to the cohorts' scientific committees.
